# Pharmacological Targeting of Phosphoinositide Lipid Kinases and Phosphatases in the Immune System: Success, Disappointment, and New Opportunities

**DOI:** 10.3389/fimmu.2012.00226

**Published:** 2012-08-02

**Authors:** Matthew D. Blunt, Stephen G. Ward

**Affiliations:** ^1^Inflammatory Cell Biology Laboratory, Department of Pharmacy and Pharmacology, University of BathBath, UK

**Keywords:** activators, inflammation, inhibitors, leukemia, lymphocytes, PI3K, SHIP-1, SHIP-2

## Abstract

The predominant expression of the γ and δ isoforms of PI3K in cells of hematopoietic lineage prompted speculation that inhibitors of these isoforms could offer opportunities for selective targeting of PI3K in the immune system in a range of immune-related pathologies. While there has been some success in developing PI3Kδ inhibitors, progress in developing selective inhibitors of PI3Kγ has been rather disappointing. This has prompted the search for alternative targets with which to modulate PI3K signaling specifically in the immune system. One such target is the SH2 domain-containing inositol-5-phosphatase-1 (SHIP-1) which de-phosphorylates PI(3,4,5)P_3_ at the D5 position of the inositol ring to create PI(3,4)P_2_. In this article, we first describe the current state of PI3K isoform-selective inhibitor development. We then focus on the structure of SHIP-1 and its function in the immune system. Finally, we consider the current state of development of small molecule compounds that potently and selectively modulate SHIP activity and which offer novel opportunities to manipulate PI3K mediated signaling in the immune system.

## Introduction

Studies using mice in which the genes encoding PI3Kδ or PI3Kγ have been either altered to encode kinase-inactive mutants (e.g., PI3Kδ^D910A^ mice) or deleted, have revealed that PI3Kδ and PI3Kγ have non-redundant (but often co-ordinated), functions in B cells, T cells, NK cell, neutrophils, mast cells, and dendritic cells (Vanhaesebroeck et al., 2005; Crabbe et al., 2007; Randis et al., 2008; Saudemont et al., 2009; Ward and Marelli-Berg, 2009). Indeed, when the immune system of these mice is challenged they exhibit severely defective responses to infection (Vanhaesebroeck et al., 2005; Crabbe et al., 2007; Ward and Marelli-Berg, 2009). The predominant expression of the γ and δ isoforms of PI3K in cells of hematopoietic lineage prompted speculation that inhibitors of these isoforms could offer selective targeting of PI3K in the immune system in a range of inflammatory and autoimmune diseases as well as in transplantation and hematological malignancies. While there has been some success in developing PI3Kδ inhibitors, progress in developing selective inhibitors of PI3Kγ has been rather disappointing. This has prompted the search for alternative targets with which to modulate PI3K signaling specifically in the immune system. In this regard, attention has recently focused on the lipid phosphatase SH2 domain-containing inositol-5-phosphatase (SHIP), which de-phosphorylates PI(3,4,5)P_3_ at the D5 position of the inositol ring to create PI(3,4)P_2._ This review will focus predominantly on the role of SHIP as a potential therapeutic target in the immune system and consider progress in developing small molecule drugs that target this protein.

## Development of Inhibitors Targeting PI3Kγ and PI3Kδ – the Story so Far

There have been huge advances in the design of PI3K inhibitors which utilize the ATP-binding pocket of PI3K to achieve greater potency and selectivity as well as reduced toxicity (Walker et al., 2000; Knight et al., 2006; Berndt et al., 2010). The development of PI3K inhibitors with which to treat cancers has made substantial recent progress (for in depth reviews on this subject see Marone et al., 2008; Workman et al., 2010; Fruman and Rommel, 2011; Shuttleworth et al., 2011; So and Fruman, 2012). However, the development of PI3K inhibitors to treat inflammatory disorders has to date, been less successful.

**Figure 1:**
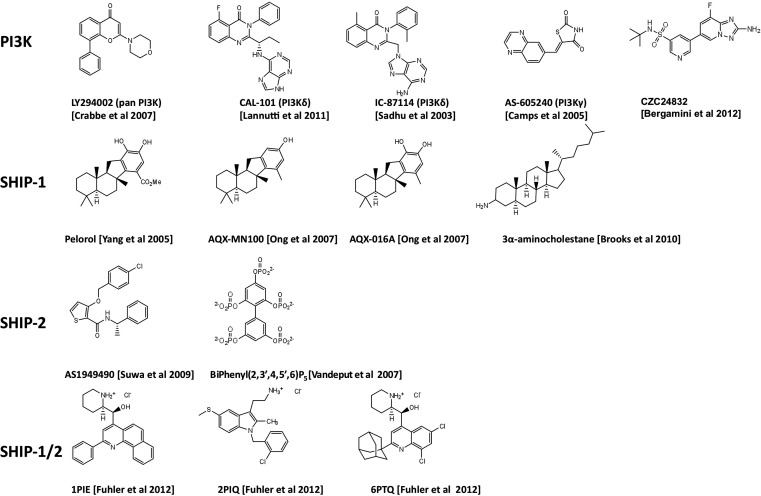
**Chemical structures of PI3K inhibitors and SHIP-targeting compounds**.

The discovery of the quinazolinone purine series, exemplified by the ICOS compound IC-87114 (Figure 1) demonstrated that the design of isoform-selective PI3K inhibitors with at least 50-fold potency over other isoforms was possible to achieve (Sadhu et al., 2003). In 2006 several members of ICOS Corporation formed a spin-out company, Calistoga Pharmaceuticals. Calistoga developed CAL-101, a PI3Kδ specific inhibitor that exhibits 40–300-fold selectivity over other PI3K isoforms. CAL-101 which was acquired by Gilead in February 2011 and recently renamed GS-1101, has shown success in clinical trials for treatment of B cell malignancies where it causes rapid lymph node shrinkage and lymphocytosis (Fruman and Rommel, 2011; Hoellenriegel et al., 2011; Lannutti et al., 2011; So and Fruman, 2012). CAL-101 displays a dual mechanism of action whereby it both decreases cell survival and reduces chemokine-mediated interactions that retain CLL cells in protective tissue microenvironments (Hoellenriegel et al., 2011; Lannutti et al., 2011). These effects have been observed across a broad range of other immature and mature B cell malignancies including CD5^+^ mantle zone B cell lymphomas, follicular lymphomas, and multiple myeloma (Herman et al., 2010; Ikeda et al., 2010; Fruman and Rommel, 2011; Hoellenriegel et al., 2011; Lannutti et al., 2011). Hodgkin lymphoma (HL) is a malignant lymphoma of B-cell origin. The malignant cells, known as Reed-Sternberg (RS) cells, represent less than 2% of the tumor mass, the remainder composed of a mix of reactive inflammatory cells attracted by the RS cells. Recently Hodgkin Lymphoma (HL) cell lines and primary samples from patients with HL have been reported to express high level of PI3Kδ and constitutive PI3K pathway activation (Meadows et al., 2012). As with CLL, CAL-101 was able to reduce the positive interaction between stromal cells and malignant RS cells. This inhibitor has therefore, demonstrated an essential role for PI3Kδ in constitutive PI3K signaling that is required for the survival of malignant B cells. Oncogenic mutations of components of the PI3K signaling pathway are infrequent in B cell malignancies. A potential mechanism for PI3K activation in this setting is tonic antigen-independent B cell receptor (BCR) signaling that requires PI3Kδ for the transduction of proliferation and survival signals.

Inhibition of the PI3Kδ isoform for the treatment of inflammatory disorders is also being explored. Specifically, CAL-101 and CAL-263 have entered clinical trials for allergic rhinitis (Table 1). In addition, patents have been filed by several other companies (Amgen, Intellikine, and Incyte) describing PI3Kδ inhibitors and the majority are based on the same basic pharmacophore identified by ICOS (Norman, 2011). However, additional scaffolds have now been reported by several companies; almost all of these are with intended indications against B cell lymphomas (Norman, 2011).

**Table 1:** **Clinical trials status of PI3K and SHIP-1 targeting compounds for the treatment of inflammatory disorders**.

Compound	Inflammatory disorder	Protein target	Clinical trial phase	Status of clinical trial	Company	Reference
AQX-1125	Asthma	SHIP-1	IIa	Initiated	Aquinox Pharmaceuticals	http://www.aqxpharma.com/content/aquinox-pharmaceuticals-initiates-two-phase-iia-clinical-studies-airway-inflammation
CAL-101	Allergic rhinitis	PI3Kδ	I	Completed	Gilead Sciences	http://clinicaltrials.gov/ct2/show/NCT00836914
CAL-263	Allergic rhinitis	PI3Kδ	I	Completed	Gilead Sciences	http://clinicaltrials.gov/ct2/show/NCT01066611
IPI-145	Inflammatory disorders	PI3Kδ/γ	I	Initiated	Infinity and Intellikine	http://www.intellikine.com/pipeline/ipi145.html

Whilst there has been considerable success in designing PI3Kδ-selective inhibitors with promise against lymphoid malignancies, the progress in designing PI3K inhibitors for anti-inflammatory/autoimmune applications has been disappointing. Compounds that selectively inhibit PI3Kγ have been identified, with a series of compounds designed by Merck Serono SA based on the thiazolidinedione scaffold (Ruckle et al., 2006). One of these, AS-605240 (Figure 1), exhibited superior potency compared to related compounds, can be administered orally and has high cell membrane permeability (Barber et al., 2005). These class of compounds have been proven useful as experimental tools but do not have requisite drug-like properties and have limited selectivity over other class 1A PI3K isoforms. Possible reasons for the relatively slow progress in developing PI3Kγ inhibitors include the close structural conservation of class I PI3Ks and other lipid kinases in the ATP-binding pocket and the limited ability of the commonly used *in vitro* assays based on recombinant enzymes, to predict cellular and *in vivo* kinase selectivity. However, Cellzome recently described a chemoproteomics-based drug discovery platform that enables multiplexed high-throughput screening of native proteins in cell extracts. The chemoproteomic approach preserves post-translational modifications and protein interactions and hence allows targeting of PI3K proteins under close-to-physiological conditions in human primary cells (Bergamini et al., 2012). Using affinity enrichment of target kinases afforded by immobilized ATP-competitive lipid kinase inhibitors, the potency of small molecule test compounds was evaluated in competition binding assays. This revealed CZC24832 which exhibits superior selectivity for PI3Kγ than previously reported compounds (Camps et al., 2005; Bergamini et al., 2012). Interestingly, CZC24832 shows anti-inflammatory effects in a collagen-induced arthritis model that correlated with reduced Th17 differentiation, a pro-inflammatory helper T cell type characterized by expression of the cytokine IL-17 (Weaver and Murphy, 2007). Indeed, CZC24832 treatment also led to reduced IL-17 production (Bergamini et al., 2012). This confirms the long-held belief that pharmacological inactivation of PI3Kγ alone, can lead to amelioration of inflammatory disease. This recent breakthrough, may facilitate detailed mechanistic studies of PI3Kγ in human primary cells and allow human clinical studies in inflammation.

The non-redundant and often co-ordinated roles of PI3Kδ and PI3Kγ in immune cell function have been reported (Rommel et al., 2007) and provide a rationale for targeting both isoforms simultaneously with a single compound. Indeed, TargeGen described two diaminopteridine-diphenol-based compounds with good selectivity for PI3Kγ and PI3Kδ that showed early promise in animal models of myocardial ischemia as well as asthma and chronic obstructive pulmonary disease (Doukas et al., 2006, 2009). The TargeGen compounds did not progress beyond phase I/II clinical trails. However, Infinity and Intellikine are currently in pre-clinical trials with IPI-145 (Table 1), which is the only PI3Kγ/δ inhibitor currently in development for the treatment of inflammatory disease (Norman, 2011).

There is an increasing appreciation of a role for PI3Kβ in the immune system including cooperation with PI3Kδ in the generation of reactive oxygen species (ROS) in neutrophils in response to fungal infection or immune complexes (Boyle et al., 2011; Kulkarni et al., 2011). Signaling responses of several Gi-coupled receptors including those for the leukocyte chemoattractants C5a and fMLP has been demonstrated to occur at least in part via PI3Kβ (Guillermet-Guibert et al., 2008). Indeed, loss of PI3Kβ confers substantial protection in a mouse model of a human autoimmune blistering disease (Boyle et al., 2011; Kulkarni et al., 2011). Loss of PI3Kβ also partially (but significantly), protected against the development of clinical signs of arthritis in response to low does of arthritogenic serum in the K/BxN mouse model of rheumatoid arthritis. However, no protection was seen mice lacking either PI3Kβ or expressing kinase-dead PI3Kδ subjected to higher does of arthritogenic serum. Remarkably, mice lacking both PI3Kβ and PI3Kδ activity were highly protected at both high and low doses of K/BxN serum. Collectively, these data provide a rationale for targeting PI3Kβ as well as PI3Kδ in the treatment of inflammatory disorders. Such dual isoform inhibitors could offer some benefit in certain therapeutic settings, though it is important to recognize that the pathogenesis of human inflammatory diseases such as RA is complex and multi-factorial. As such, the precise contribution of each isoform to disease pathology is likely to be subtle and complex. Nevertheless, compounds with dual selectivity for PI3Kβ and PI3Kδ have been reported suggesting that this approach is feasible (Knight et al., 2006). However, caution should be applied to the use of PI3Kβ inhibitors in inflammatory disorders due to the described role of PI3Kβ in thrombus formation and circulatory homeostasis (Bird et al., 2011).

## Increased Understanding of a Role for Other PI3KS in the Immune System

The difficulties of developing PI3Kγ inhibitors with sufficient selectivity over PI3K isoforms has led to the search for other targets that might offer opportunities to selectively disrupt PI3K signaling in immune cells. To this end, class II PI3KC2β, has been demonstrated to play an important and unexpected role in CD4^+^ T-cell activation downstream of the TCR (Srivastava et al., 2009), while Vps34’s role in autophagy (Backer, 2008; Simonsen and Tooze, 2009), suggests it may prove important for immune recognition of tumor antigens, regulation of T cell homeostasis, and immune tolerance (Li et al., 2008; Nedjic et al., 2008; Walsh and Edinger, 2010). There is considerable evidence that class III PI3K is important for phagocytosis (Fratti et al., 2001; Vieira et al., 2001; Ellson et al., 2006; Anderson et al., 2008). There may be opportunities to target Vps34 in destructive inflammatory/autoimmune diseases where there is dysregulated phagosomal activity and antigen presentation of self molecules, for example. The publication of the Vps34 crystal structure in complex with PI3K inhibitors may allow the design of more potent and selective Vps34 inhibitors which are able to exploit differences between Vps34 and Class 1 PI3Ks (Miller et al., 2010). However, the largely ubiquitous expression of Class II and III PI3Ks makes selective targeting of the immune system problematic.

## SHIP-1: An Alternative Target for Modulation of PI3K Signaling in the Immune System

The search for alternative targets with which to modulate PI3K signaling specifically has therefore, recently focused on the lipid phosphatase SH2 domain-containing inositol-5-phosphatase-1 (SHIP-1), which de-phosphorylates PI(3,4,5)P_3_ at the D5 position of the inositol ring to create PI(3,4)P_2_. The *INPP5D* gene located on chromosome 2 (2q37.1) encodes the 145-kDa SHIP-1 which was originally recognized as an important component of the inhibitory signaling pathway triggered by the IgG receptor FcγRIIB in mast cells and B cells (Ono et al., 1996). Once recruited to the plasma membrane by signaling complexes, its catalytic activity depletes PI(3,4,5)P_3_ and prevents membrane localization of some PH domain-containing effectors, leading to inhibition of extracellular calcium influx and ultimately reducing transcription activation, and cytokine release. One would predict that activators of SHIP-1 would lead to a reduction of cellular PI(3,4,5)P_3_ levels and hence, mimic the effect of PI3K inhibitors. Its hematopoietic-restricted expression should limit the impact of SHIP-1 targeted drugs to the immune system making SHIP-1 an attractive drug target for use in inflammatory and autoimmune diseases, hematological malignancies as well as in transplantation settings.

## SHIP-1: A Crossroads in PI3K-Dependent Signaling

**Figure 2:**
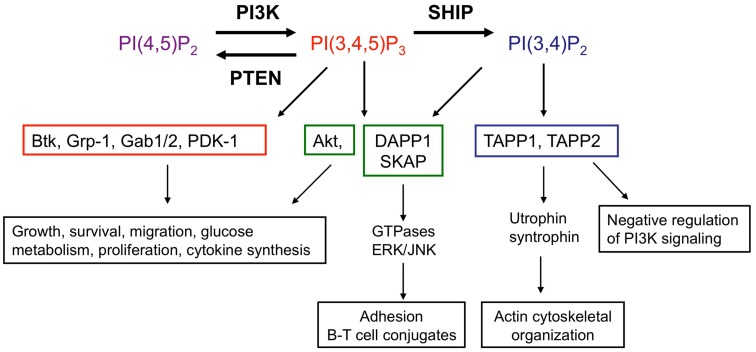
**SHIP acts as a molecular “switch.”** SHIP catalyzes the conversion of the PI3K lipid product PI(3,4,5)P_3_ to PI(3,4)P_2_. Effector proteins which express PH domains are recruited and activated by these lipid second messengers at the cell surface membrane. PH domains of proteins are able to discriminate between PI(3,4,5)P_3_ and PI(3,4)P_2_. Examples of proteins which bind only PI(3,4,5)P_3_ (red), both PI(3,4,5)P_3_ and PI(3,4)P_2_ (green), or only PI(3,4)P_2_ (blue) as well as functional consequences are shown, though there are many other PH domain proteins present in immune cells and this is not an exhaustive list. Functional read-outs downstream of PI(3,4,5)P_3_-interacting proteins and Akt are context dependent, have been extensively reviewed elsewhere (Manning and Cantley, 2007;Vanhaesebroeck et al., 2010, 2012) and are summarized in this figure. Lesser known interacting partners of PI(3,4)P2-dependent TAPP-1/2 and PI(3,4)P2/PI(3,4,5)P_3_-dependent DAPP1 with roles in immune function are indicated (Costantini et al., 2009; Zhang et al., 2009; Vanhaesebroeck et al., 2010; Wullschleger et al., 2011; So and Fruman, 2012). Abbreviations: Btk, Bruton’s tyrosine kinase; Gab1, GRB2-associated binding protein-1; Grp-1, general receptor for phosphoinositides 1; PDK-1, phosphoinositide lipid-dependent kinase-1; DAPP1, dual-adapter for phosphotyrosine and 3-phosphoinositides 1; SKAP, src kinase-associated phosphoprotein; TAPP, tandem pleckstrin homology domain protein.

The classical view of SHIP-1 is that it acts to switch off PI3K-dependent signaling by degradation of PI(3,4,5)P_3_. However, the metabolism of PI(3,4,5)P_3_ by SHIP-1 yields PI(3,4)P_2_ which retains the phosphate grouping on the third position of the inositol ring and thus, may retain some signaling ability (Figure 2). Pleckstrin homology (PH) domains encoded in many proteins (e.g., Grp-1, Gabs, and Btk) bind exclusively to PI(3,4,5)P_3_, whereas others such as those found in dual adaptor of phosphotyrosine and 3-phosphoinositides-1 (DAPP1) and Src kinase-associated phosphoprotein (SKAP), can interact with both PI(3,4,5)P_3_ and PI(3,4)P_2_ (Lemmon and Ferguson, 2000; Zhang et al., 2009). In addition, the tandem PH domain-containing protein TAPP-1 encodes PH domains that show selectivity toward PI(3,4)P_2_ (Dowler et al., 2000). The ability of PH domain-containing proteins to distinguish between different 3′-phosphoinositide lipids suggests that SHIP-1 can act as a switch to redirect PI3K-dependent signaling toward a set of distinct effectors that are temporally and functionally separate from PI(3,4,5)P_3_-dependent events. Thus, SHIP-1 may function to fine-tune phosphoinositide signaling, rather than terminate it. In this regard, SHIP-1 promotes recruitment of the GTPase Irgm1 to sites of phagocytosis in macrophages via generation of PI(3,4)P_2_, a critical step in maturation of the phagosome and engulfment of bacteria (Tiwari et al., 2009). PI(3,4,5)P_3_ and PI(3,4)P_2_ appear sequentially following agonist stimulation in many cell types including T lymphocytes, but show temporal overlap. Some cell types, notably B lymphocytes and platelets, exhibit sustained PI(3,4)P_2_ production, lasting for up to 45–60 min post-stimulation (Sorisky et al., 1992; Brauweiler et al., 2001).

## Non-Enzymatic Activities of SHIP-1

**Figure 3:**
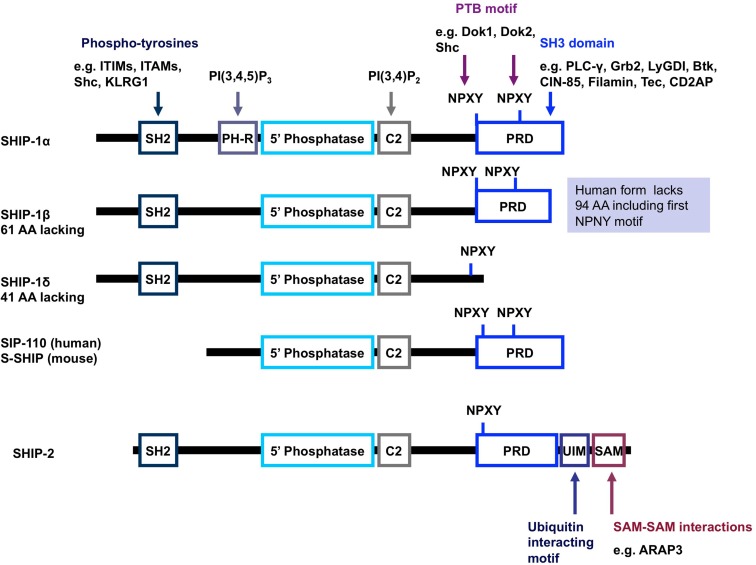
**Schematic representation of the structure of SHIP-1 and its isoforms**. SHIP-1 possesses a centrally located 5′ phosphatase catalytic domain, an SH2 domain at the N-terminus as well as a proline rich domain and NPXY motifs at the C-terminus (Harris et al., 2008). SHIP-1 also has a C2 domain adjacent to the catalytic domain which, when bound to PI(3,4)P_2_, acts to allosterically enhance the catalytic activity of SHIP (Ong et al., 2007). A pleckstrin homology-related domain that binds PI(3,4,5)P_3_ has also been reported to exist adjacent to the catalytic domain in SHIP-1 (and most likely the other forms of SHIP-1). Structures depicted represent mouse protein; key differences in human protein structure are annotated on the right-hand side (shaded blue background). Abbreviations: PH-R, pleckstrin homology-related domain; PRD, proline rich domain; SH2, src homology domain; UIM, ubiquitin interacting motif; SAM, sterile alpha motif.

SH2 domain-containing inositol-5-phosphatase-1 protein possesses numerous structural domains in addition to its single catalytic domain (Figure 3). The catalytic domain is responsible for the hydrolysis of the 5-phosphate group on the PI3K product PI(3,4,5)P_3_ to form PI(3,4)P_2_. Under basal conditions, SHIP-1 is located in the cell cytosol and upon receptor ligation is recruited to the surface membrane, bringing SHIP-1 within close proximity to its lipid substrate. Numerous structural domains are required for SHIP-1 to successfully re-localize to the surface membrane. The SH2 domain within SHIP-1 interacts with proteins via the consensus amino acid sequence pY[Y/S][L/Y/M][L/M/I/V]. Through this SH2 domain, SHIP-1 binds to tyrosine phosphorylated proteins such as Shc, Doks, Gabs, CD150, platelet-endothelial cell adhesion molecule (PECAM), Cas, c-Cbl, certain immunoreceptor tyrosine-based inhibitory motifs (ITIMs), and some immunoreceptor tyrosine-based activation motifs (ITAMs). Proline rich regions within the C-terminal enable SHIP-1 to bind proteins that contain a SH3 domain, for example phospholipase-Cγ and Grb-2. The phosphorylation of tyrosine residues within the NPXY motifs at the C-terminal tail of SHIP-1 provides sites of interaction for various proteins which express phosphotyrosine-binding (PTB) domains, such as Shc, Dok1, and Dok2. A newly identified structural domain has been recently identified whereby a segment of SHIP-1 adopts an independently folded structure predicted to have PH domain-like topology. This PH-related (PH-R) domain binds PI(3,4,5)P_3_ and is required for localization of SHIP-1 to the phagocytic cup and SHIP-1 mediated inhibition of FcγR-mediated phagocytosis by macrophages (Ming-Lum et al., 2012).

The various structural domains not only serve to bring SHIP-1 in close proximity to its substrate at the surface membrane, but also allow SHIP-1 to perform a scaffolding role, recruiting other proteins to the surface membrane independent of its catalytic activity. Most of these interactions contribute to the negative regulation of PI3K signaling by SHIP independently of its catalytic activity. For example, binding of SHIP-1 to ITAM containing adaptor proteins via the SH2 domain, can prevent PI3K recruitment via the p85 regulatory subunit (Peng et al., 2010). Moreover, SHIP-1 has also been shown to block, independently of its catalytic activity, the recruitment of the tyrosine phosphatase SHP1 to the SLAM family receptor 2B4 in NK cells (Wahle et al., 2007). Indeed, there is a profound increase in SHP1 recruitment in SHIP null cells that tips the balance toward constitutive inhibitory signaling via 2B4 (Wahle et al., 2007). Other protein interactions allow SHIP-1 to negatively influence different signaling pathways. For example, in B lymphocytes following BCR and FcγRIIB co-ligation, SHIP-1 interactions with Shc inhibit Ras/MAPK activation either by displacing Grb-2 and Sos from their interaction with Shc and/or by recruiting Dok1 and RasGAP to FcγRIIB at the plasma membrane. In T cells, SHIP-1 interacts with the Tec kinase and inhibits its function in T cells (Tomlinson et al., 2004a) and participates in a negative signaling complex comprising Grb-2-SHIP-1 and Dok1/2 that is recruited to LAT and inhibits Akt and PLCγ activation (Dong et al., 2006).

There is evidence that the scaffolding role of SHIP-1 is not restricted to facilitating negative regulatory mechanisms. For example, adaptor functions of SHIP-1 potentiate EGF-induced PLC-gamma1 activation in COS cells over-expressing SHIP-1 (Song et al., 2005). In addition, SHIP-1 facilitates a positive regulatory role in TLR-induced cytokine production from mucosal mast cells (Ruschmann et al., 2012). Recently, SHIP-1 has been reported to interact via its proline rich region with the Cbl-interacting 85-kDa protein CIN85 (Buchse et al., 2011) and the related CD2-associated adaptor protein (CD2AP) in Bao et al. (2012). In T cells, CIN85 binds to the adaptor molecule SH3 domain-binding protein-2 (3BP2), which is involved in leukocyte signaling downstream of Src/Syk-kinase coupled immunoreceptors and formation of the immunological synapse (Le Bras et al., 2007), though its role in B cells is unclear. In plasmacytoid dendritic cells, the CDAP-SHIP-1 complex positively regulates BDCA2/FcεR1γ signaling by inhibiting Cbl-mediated ubiquitination and degradation of the activated Syk and FcεR1γ in plasmacytoid dendritic cells (Bao et al., 2012). Finally, it appears that the SH2 domain of SHIP-1 interacts (both intra- and inter-molecularly) with phosphorylated NPXY^1020^ within the SHIP-1 C-terminus that leads to dimerization and oligomerization (Mukherjee et al., 2012). SHIP-1 lacking its C-terminus is activated 8–10-fold more than full length SHIP-1 (Zhang et al., 2010), suggesting that the C-terminus not only controls interactions of SHIP-1 with respective binding partners, but also catalytic activity of SHIP-1.

## Role of SHIP-1 in Regulating Immune Function

SH2 domain-containing inositol-5-phosphatase-1 is recruited to the surface membrane following ligation of a variety of receptors including, chemokine, Toll-like receptors (TLR), antigen, co-stimulatory, and cytokine receptors as well as IgG engagement by FcγRIIB (Harris et al., 2008; Keck et al., 2010; Table 2). SHIP-1 knock-out mice have proven invaluable in identifying the crucial role of SHIP-1 in the regulation of mast cell degranulation, BCR signaling and auto-antibody production, dendritic cell function, and NK cell cytolytic function (Table 3). SHIP-1 also regulates TLR signaling (Sly et al., 2004; Gabhann et al., 2010; Ruschmann et al., 2012), leukocyte polarization, and migration (Nishio et al., 2007; Harris et al., 2011; Mondal et al., 2012). It has also been shown to play a central role in CD4-mediated inhibitory signaling activated by HIV-1 gp120 that leads to disarmament of the immune systems (Waterman et al., 2012). Remarkably, there is evidence that SHIP is able to influence PI3K signaling not only at receptors it is recruited to (in *cis*) but also at other receptors where it is not recruited directly (in *trans*; Brauweiler et al., 2007; Fortenbery et al., 2010). For example, CXCL12/CXCR4-induced calcium mobilization and cell migration is impaired by prior activation of FcγRIIB and this inhibition is reduced in SHIP-deficient B cells (Brauweiler and Cambier, 2003; Brauweiler et al., 2007). Consistent with a role for SHIP-1 in inhibition, signaling through CXCR4 by CXCL12 is dependent on PI(3,4,5)P_3_ (Brauweiler et al., 2007). Similarly, SHIP-1 acting in *trans* from the 2B4 has been proposed to oppose PI3K activity at other receptors such as the MHC-I receptor in NK cells (Fortenbery et al., 2010).

**Table 2:** **Key receptors in the immune system that are known to recruit and/or be regulated by SHIP-1**.

Receptor	Reference
B cell receptor	Okada et al. (1998)
CD16	Galandrini et al. (2002)
CD22	Poe et al. (2000)
CD28	Edmunds et al. (1999)
TCR/CD3 complex	Dong et al. (2006) and Osborne et al. (1996)
CXCR4	Wain et al. (2005)
FcεR1	Gimborn et al. (2005), Huber et al. (1998), and Kimura et al. (1997)
FcγRIIa	Nakamura et al. (2002)
FcγRIIb	Ono et al. (1996)
Granulocyte colony-stimulating factor receptor	Hunter and Avalos (1998)
IL-3 receptor	Liu et al. (1999)
KLRG1	Tessmer et al. (2007)
TLR2	Keck et al. (2010)
TLR3	Gabhann et al. (2010) and Ruschmann et al. (2012)
TLR4	Keck et al. (2010) and Sly et al. (2004)
TLR9	Ruschmann et al. (2012)
2B4	Wahle et al. (2007)

*See main text for further details regarding whether SHIP-1 mediated negative or positive impact on signal transduction events elicited via each receptor*.

**Table 3:** **Impact of SHIP-1 gene targeting on leukocytes**.

Cell type	Phenotype of SHIP-1 KO
Basophils	SHIP-1^−/−^ mice show increased Th2 skewing due to increased IL-4 secretion from basophils (Kuroda et al., 2011)
B cell	Btk membrane association increased. Hyper-responsive to cross-linking of BCR (Bolland et al., 1998; Helgason et al., 2000)
	Loss of anergy, production of auto-antibodies (O’Neill et al., 2011)
Dendritic cell	Enhanced survival and proliferation, but impaired maturation (Antignano et al., 2010)
	Reduced nitric oxide production; SHIP-1 null DC’s suppress T cell proliferation (Antignano et al., 2011)
Mast cell	Enhanced maturation of BMMC, CTMC, and MMC; reduced IgE-induced BMMC survival; enhanced degranulation of BMMCs, CTMC, and MMC (Kalesnikoff et al., 2002; Ruschmann et al., 2012)
	Enhanced TLR expression and TLR-induced cytokine production from CTMCs via adaptor-mediated pathway (Ruschmann et al., 2012)
Myeloid cell	Increased myeloid suppressor cell numbers (Ghansah et al., 2004)
	Increased M2 macrophage skewing (indirect mechanism via increased IL-4 secretion from basophils; Kuroda et al., 2009)
	Increased ratio of PI(3,4,5)P_3_ to PI(3,4)P_2_ on phagosomal membrane. Decreased early NADPH oxidative activity in phagosomes (Kamen et al., 2008)
Natural killer cells	Deficient receptor repertoire. Defective IFNγ secretion. Increase in peripheral number. Defective cytolytic function (Fortenbery et al., 2010)
T cell	Increased regulatory T cell differentiation, decreased Th17 development (Locke et al., 2009)
	Enhanced Th1 differentiation and CD8 cytotoxic activity. Decreased Th2 differentiation (Tarasenko et al., 2007)^a^

*N.B data is derived from germline SHIP-1 knock-out cells, except where denoted*.

*^a^The reported phenotype is derived from a T cell-specific SHIP-1 knock-out*.

*BMMC, bone marrow-derived mast cells; CTMC, connective tissue mast cells; MMC, mucosal mast cells*.

It is also clear that SHIP-1 has a pivotal role in regulating the balance between pro-inflammatory and anti-inflammatory myeloid and lymphoid cells (Ghansah et al., 2004; Locke et al., 2009; Kuroda et al., 2011). For example, SHIP-1 deficient mice exhibit more myeloid-derived suppresser cells (MDSCs) than their wild type counterparts (Ghansah et al., 2004). Selective ablation of SHIP expression in either myeloid or T lymphoid lineage cells, has revealed that myeloid-specific ablation of SHIP leads to the expansion of both MDSC and Treg-cell numbers, indicating SHIP-dependent control of Treg-cell numbers by a myeloid cell type. Conversely, T-lineage specific ablation of SHIP leads to expansion of Treg-cell numbers, but not expansion of the MDSC compartment, indicating SHIP also has a lineage intrinsic role in limiting Treg-cell numbers (Collazo et al., 2012). G-CSF is required for expansion of the MDSC splenic compartment in mice rendered SHIP-deficient as adults. Thus, SHIP controls MDSC numbers, in part, by limiting production of the myelopoietic growth factor G-CSF (Collazo et al., 2012).

SH2 domain-containing inositol-5-phosphatase-1 also plays a role in regulating the balance of M1 macrophages (implicated in the first inflammatory response) and M2 macrophages (implicated in inflammatory response termination, tissue repair, regeneration, and remodeling). SHIP-1 deficiency leads to increased macrophage skewing toward M2 macrophages. This indicates that PI(3,4,5)P_3_ drives macrophage progenitors toward an M2 phenotype and that SHIP-1 blocks this skewing (Rauh et al., 2005; Kuroda et al., 2009). Moreover, SHIP-1 is essential for normal Th17 cell development and plays a key role in the reciprocal regulation of Tregs and Th17 cells (Collazo et al., 2009; Locke et al., 2009). Germline SHIP deficiency promotes a preferential expansion and/or accumulation of conventional Tregs that have increased expression of FoxP3 indicating that SHIP limits Treg-cell function *in vivo* and limits FoxP3 acquisition by naïve CD4^+^ T cells (Collazo et al., 2009). Mice carrying a T cell-specific deletion of SHIP-1 uncovered a regulatory role for SHIP-1 in controlling Th1/Th2 bias and cytotoxic responses as a result of its inhibitory effect on T-bet expression. Hence, SHIP-1 null T cells do not skew efficiently to a Th2 phenotype and display Th1-dominant immune responses *in vitro* and *in vivo* (Tarasenko et al., 2007). This is in contrast to evidence from germ line SHIP-1 null mice, which indicates that SHIP-1 can also repress Th2 skewing by inhibiting IL-4 production from basophils (Kuroda et al., 2011).

T cell-specific deletion of SHIP-1 using CD4CreSHIP^flox/flox^ mice, had no affect on T-cell development, activation state, or Treg-cell numbers (Tarasenko et al., 2007). However, a recent study using in LckCreSHIP^flox/flox^ mice reported significant reduction in the frequency of splenic CD3^+^ T cells and CD4^+^ and CD8^+^ T cells in the peripheral blood relative to SHIP^flox/flox^ controls (Collazo et al., 2012). The discrepancy may be because deletion of SHIP in CD4CreSHIP^flox/flox^ mice may occur at a different time point during T-cell development compared to SHIP deletion in LckCreSHIP^flox/flox^ mice.

## The SHIP Navy: A Force for Diversity and Complexity of Function

Multiple forms of the INPP5D gene product can occur via post-translational modification, degradation, or alternative mRNA splicing. This produces SHIP-1 proteins of 145 kDa (SHIPα), 135 kDa (SHIPβ), and 110 kDa (SHIPδ) in size. In addition, other 130, 125, and 110 kDa forms of SHIP-1 have been reported (Hamilton et al., 2011; Kerr, 2011). Truncated SHIP-1 proteins exhibit differential protein binding properties owing to the lack of/altered expression of certain protein binding domains (Figure 3). For example, s-SHIP and its human homolog SIP-110, are truncated at the N-terminus and lack the SH2 domain, but retain the catalytic, C2 and proline rich domains. This limits the repertoire of binding proteins available for interaction and hence, these forms cannot interact with Shc, yet can still interact with Grb-2. Moreover, s-SHIP is mostly localized at the plasma membrane rather than the cytoplasm (Hamilton et al., 2011; Kerr, 2011). Although originally thought to be restricted to embryonic stem cells, s-SHIP expression has been reported in adult hematopoietic cells and synergizes with SHIP-1 to regulate the activation of macrophages (Nguyen et al., 2011).

SHIP-2 is a 142-kDa protein encoded by a separate gene, yet still retains approximately 65% homology with SHIP-1 within the catalytic domain. Divergence between SHIP-1 and SHIP-2 occurs largely within the proline rich domains as well as within the SH2 domain. In addition, SHIP-2 contains a unique sterile alpha motif (SAM) domain that can be involved in SAM–SAM domain interactions with other proteins, for example ARAP3 (Raaijmakers et al., 2007). SHIP-2 also shows the presence of an ubiquitin interacting motif at the C-terminal end and (unlike SHIP-1), it can hydrolyze PI(4,5)P_2_ *in vitro*. SHIP-2 expression is not restricted to hematopoietic cell lineages and can be detected in heart, skeletal muscle, and brain tissues. SHIP-2 appears to have a major role in the negative regulation of insulin signaling in non-immune cells (Ooms et al., 2009). SHIP-1 and SHIP-2 are co-expressed in T cells and both are potent negative regulators of PI(3,4,5)P_3_-mediated signals (Bruyns et al., 1999; Brauweiler et al., 2001). Tyrosine phosphorylation of SHIP-2 in T lymphocytes has not been reported, but it may still be enzymatically active and hence, the SHIP-1 knock-outs may have an incomplete phenotype. Interestingly, it is becoming clear that although SHIP-1 and SHIP-2 can interact with common binding partners, they additionally have their own unique profile of interacting partner proteins (Table 4), that possibly reflects the differences in their non-catalytic domains (Erneux et al., 2011; Mehta et al., 2011).

**Table 4:** **SHIP-1 and SHIP-2 interacting proteins**.

SHIP-1 interacting proteins	SHIP-2 interacting proteins	SHIP-1 and SHIP-2 interacting proteins
CD2AP (Bao et al., 2012)	Actin, non-muscle^a^ (Mehta et al., 2011)	Btk^d^ (Tomlinson et al., 2004b; Xie et al., 2008a)
Ezrin, Radaxin, and Meosin^a^ (Mehta et al., 2011)	APS^a^ (Onnockx et al., 2008)	CIN-85^a,c^ (Havrylov et al., 2009; Buchse et al., 2011)
FUBP2^a^ (Mehta et al., 2011)	ARAP3^b^ (Raaijmakers et al., 2007)	DOk1a (Tamir et al., 2000; Havrylov et al., 2009; Cunningham et al., 2010)
Grb-2^a^ (Mehta et al., 2011)	c-Cbl^b^ (Vandenbroere et al., 2003)	Filamin^a,c^ (Dyson et al., 2001; Lesourne et al., 2005)
KLRG1^a^ (Tessmer et al., 2007)	Glucose-regulated protein precursor^a^(Mehta et al., 2011)	Shc^a^ (Mehta et al., 2011)
LyGDI^a^ (Mehta et al., 2011)	Heat shock protein 90-beta^a^ (Mehta et al., 2011)	Tec^d^ (Tomlinson et al., 2004a)
PKC-δ^a^ (Chari et al., 2009)	Hematopoietic cell specific Lyn substrate^a^(Mehta et al., 2011)	
PLC-γ1^a^ (Song et al., 2005)	HSP90β^a^ (Mehta et al., 2011)	
	Intersectin 1^b^ (Xie et al., 2008b)	
	Protein disulfide-isomerase A3 precursor^a^ Mehta et al., 2011)	
	PR130^c^ (Zwaenepoel et al., 2010)	
	PTP1B^c^ (Mertins et al., 2008)	
	p130Cas^a^ (Prasad et al., 2001)	
	JIP1^b^ (Xie et al., 2008a)	
	Tubulin beta-2A chain^a^ (Mehta et al., 2011)	
	Vinexin^c^ (Paternotte et al., 2005)	

*Methods by which interactions have been identified are indicated: ^a^immunoprecipitation; ^b^yeast two hybrid; ^c^mass spectrometry; ^d^GST-SH3 pull-down. Where known, the domains within SHIP-1 that interact with these proteins are indicated in Figure 3. Protein interactions with SHIP-1 that are known to result in positive signaling outcomes are indicated in red. Refer to main text for further detail*.

*ARAP3, Arf-GAP, Rho-GAP, ankyrin repeat and PH domain-3; CIN85, Cbl-interacting 85 kDa protein; FUBP2, far upstream element binding protein-2; JIP1, c-Jun NH_2_ terminal kinase (JNK)-interacting protein-1; KLRG1, killer cell lectin-like receptor G1; LyGDI, a Rho guanine nucleotide dissociation inhibitor originally identified in lymphocytes; PTPB1, protein-tyrosine phosphatase 1B*.

## The Role of SHIP-1 in Hematological Malignancies

Over-activation of PI3K-dependent signaling cascades is a common occurrence in many human cancers (Engelman, 2009). The lipid phosphatase PTEN which also negatively regulates PI(3,4,5)P_3_ accumulation by de-phosphorylating the D3 position of the inositol ring, is a well characterized tumor suppressor gene (Hollander et al., 2011). Likewise, evidence for mutations of SHIP-1 have also been shown in acute lymphoblastic leukemia (Luo et al., 2003) and in acute myeloid leukemia (Luo et al., 2004).

The loss of SHIP-1 has also been shown to promote the development of erythroleukemia, with SHIP-1 identified as a target gene of the oncogene fli-1 (Lakhanpal et al., 2010). There are at least two mechanisms by which SHIP-1 expression may be down-regulated. The first involves targeting of SHIP-1 by miR-155 in B cells, where high levels of miR-155 and reduced SHIP-1 expression have been linked to the development of acute lymphoblastic leukemia in mice (Costinean et al., 2009). miR-155 levels were also found to be significantly increased in human patients with diffuse large B cell lymphoma (Eis et al., 2005). The second involves BCR-ABL (the oncogene responsible for chronic myeloid leukemia), which either directly or via a Src kinase family member, tyrosine phosphorylates SHIP-1. This leads to polyubiquitination of SHIP-1 and subsequent STAT6 dependent-proteasomal degradation (Ruschmann et al., 2010). Interestingly, there is an inverse relationship between expression of SHIP-1 and BCR-ABL (Martino et al., 2001; Jiang et al., 2003). Thus, reduced SHIP-1 activity might be a prerequisite for the proliferative advantage of some chronic myeloid leukemia clones. A similar inverse relationship exists between a constitutively active oncogenic c-kit receptor and SHIP-1, whereby inhibition of c-kit’s intrinsic tyrosine kinase activity with Imatinib reversibly raises SHIP-1 levels (Vanderwinden et al., 2006).

The role of SHIP-1 as a tumor suppressor is also evident in the ability of SHIP-1 to restrict myeloid suppressor cells and regulatory T cells (Ghansah et al., 2004; Locke et al., 2009). Therefore the loss of SHIP-1 expression/function may lead to increased suppression of T-cell mediated anti-tumor immunity. Indeed, in murine pancreatic cancer SHIP-1 expression was shown to be reduced in splenocytes which also correlated with an increase in myeloid suppressor cell numbers (Pilon-Thomas et al., 2011). Decreased SHIP-1 expression has also been shown in myelodysplastic syndrome progenitor cells, whereas over-expression of SHIP-1 inhibited myeloid leukemic growth (Lee et al., 2012).

The role of SHIP-1 in leukemia however, appears more complex than initially thought. For example, while PTEN can suppress growth and apoptosis, SHIP-1 was shown not to act as a tumor suppressor in myeloma cells (Choi et al., 2002). The use of a small molecule SHIP-1 inhibitor demonstrated that catalytically active SHIP-1 is required for the survival of multiple myeloma cells (Brooks et al., 2010) and that therefore, in certain cases, SHIP-1 actually supports cancer cell survival. This would be consistent with increased levels of the SHIP-1 enzymatic product PI(3,4)P_2_ promoting Akt activation and survival/proliferation (Manning and Cantley, 2007). Indeed, another group has shown that SHIP-1 inhibits CD95/Fas-mediated apoptosis of T cells, albeit independently of its catalytic activity (Charlier et al., 2010).

## SHIP-1 and the Stem Cell Niche

The crucial role of PTEN/Akt in the maintenance of stem cell homeostasis is now evident (Hill and Wu, 2009; Song et al., 2012). It is now becoming clear that SHIP-1 also has an important role in maintaining the stem cell niche. Hemopoietic stem cell (HSC) proliferation is increased in SHIP-1 null mice. Despite expansion of the compartment, SHIP-1 deficient HSCs exhibit reduced capacity for long-term repopulation and home inefficiently to bone marrow (Desponts et al., 2006; Hazen et al., 2009). The role of SHIP-1 in the biology of both HSC and the hematopoietic stem cell niche, suggests that it may be a useful target for treatment of bone marrow failure syndromes caused by viruses, radiation, chemotherapy, or malignancy. As already mentioned, MDSCs are a type of immunoregulatory cell that can repress allogeneic T cell responses. A common complication arising after bone marrow transplantation is Graft-versus-host disease (GVHD) which involves priming of allogeneic T cells. Remarkably, SHIP-1 deficient mice express more myeloid suppressor cells than their wild type counterparts and accept allogeneic bone marrow grafts with a reduced incidence of GVHD (Ghansah et al., 2004; Kerr, 2008). In addition SHIP-1 null mice are better able to accept bone marrow transplants compared to controls (Wang et al., 2002) and SHIP-1 deficient mice have shown reduced cardiac graft rejection compared to controls (Collazo et al., 2009).

## SHIP is Targeted by Pathogens to Avoid Immune Recognition

The key regulatory role of SHIP-1 has been exploited by several opportunistic pathogens that target these phosphatases in order to evade immune detection. Thus, lymphocytes are particularly sensitive to the cytolethal distending toxin subunit B (CdtB), an immunotoxin produced by *Actinobacillus actinomycetemcomitans*, that can hydrolyze PI(3,4,5)P_3_ to PI(3,4)P_2_. Exposure to CdtB leads to cell cycle arrest and death by apoptosis. The lipid phosphatase activity of CdtB may therefore, result in reduced immune function, facilitating chronic infection with *Actinobacillus* and other enteropathogens that express Cdt proteins (Shenker et al., 2007). The measles virus evades destruction by the immune system, at least in part, by targeting negative regulation of PI3K/Akt signaling. It induces expression of the SHIP-1 homolog SIP-110 which depletes cellular PI(3,4,5)P_3_ pools, suggesting that the threshold for activation signals leading to induction of T cell proliferation is raised (Avota et al., 2006). The targeting of this protein by pathogens to avoid immune recognition, emphasizes the notion that SHIP-1 might offer opportunities for the design of new drugs targeting PI3K-dependent signaling.

## Pharmacological Manipulation of SHIP

### Allosteric SHIP-1 activators

In 2005, pelorol (a product of the marine invertebrate *Dactylospongia elegans*) was described as an activator of SHIP-1 (Yang et al., 2005). More potent synthetic chemical entities have since been designed by Aquinox Pharmaceuticals (Figure 1). Along with PI(3,4)P_2_, these compounds were shown to allosterically enhance catalytic activity by binding to the C2 domain of SHIP-1 (Figure 3). The C2 domains of SHIP-1 and SHIP-2 share 38% homology (compared to 51% homology between total SHIP-1 and SHIP-2 in humans), and it is believed that this reduced homology in the C2 domain allows these pelorol based compounds to achieve SHIP-1 selectivity. This is particularly important given the crucial role of SHIP-2 in the regulation of insulin signaling (Ooms et al., 2009). Two of these compounds, AQX-016A and AQX-MN100, exhibited potent inhibition of immune cell activation *in vitro* and were anti-inflammatory *in vivo* using mouse models of endotoxemia and acute cutaneous anaphylaxis (Ong et al., 2007). Intriguingly, these SHIP-1 activating compounds increased apoptosis of multiple myeloma cells *in vitro* and when used in combination with bortezomib (an established multiple myeloma treatment) proved more effective at inhibiting cancer cell proliferation than bortezomib alone (Kennah et al., 2009). Other compounds based on the structure of pelorol have been developed by Aquinox Pharmaceuticals as SHIP-1 activating compounds with a view for application in inflammatory disorders. AQX-1125 is the most advanced and has passed Phase 1 clinical trials in 2011, with Phase IIa clinical studies initiated in late 2011 for the treatment of mild and moderate asthma (Table 1). With regard to the latter, the recent finding that TLR stimulation augments IgE plus Ag-induced TNFa and IL-6 production from MMCs (Ruschmann et al., 2012) might explain the exacerbation of IgE-mediated allergic episodes by infectious agents (Qiao et al., 2006). Since IgE synergizes with TLR ligands to trigger cytokine production from SHIP-1 null mucosal mast cells, activating SHIP-1 specifically in these cells might be useful for treating chronic inflammatory diseases like asthma.

### SHIP-1 inhibitors

A novel small molecule selective inhibitor of SHIP-1, termed 3 α-aminocholestane (3AC, Figure 1) has also recently been identified using high-throughput screening, though the site of drug-protein interaction is unclear (Brooks et al., 2010). Consistent with observations from SHIP-1 deficient mice, treatment of mice with 3AC led to increased numbers of myeloid suppressor cells and reduced ability of peripheral lymphoid tissues to prime myeloid-associated responses and protected against GVHD (Brooks et al., 2010). The inhibition of SHIP-1 using pharmacological compounds may therefore offer the potential to aid transplant acceptance in patients undergoing transplant surgery. 3AC also increased levels of granulocytes, red blood cells, neutrophils, and platelets in mice and could therefore, have potential to improve blood cell number in patients with myelodysplastic syndrome or myelosuppressive infection.

Remarkably, SHIP-1 inhibition using 3AC induced the apoptosis of human acute myeloid leukemia cell lines which is consistent with SHIP-1 being anti-apoptotic under some circumstances (Brooks et al., 2010). Further studies showed that 3AC inhibited multiple myeloma cell growth in a tumor xenograft model in mice (Fuhler et al., 2011). Since both substrate [PI(3,4,5)P_3_] and product [PI(3,4)P_2_] of SHIP-1 have been shown to influence Akt activation and cell survival, this may explain in part, why both activators and inhibitors of SHIP-1 have shown efficacy against leukemic cells (Kerr, 2011).

### SHIP-2 inhibitors

SHIP-2 is thought to be involved in type-2 diabetes and obesity (Ooms et al., 2009) as well as cancer and atherosclerosis (Suwa et al., 2010). The development of compounds which selectively target SHIP-2 has therefore been of great interest. Small molecule compounds which specifically inhibit the catalytic activity of SHIP-2 have recently been described (Suwa et al., 2009). In addition a novel biphenyl 2,3′4,5′,6-pentakisphosphate [BiPh(2,3′,4,5′,6)P_5_] compound has demonstrated potent inhibition of SHIP-2 catalytic activity (Vandeput et al., 2007). BiPh(2,3′,4,5′,6)P_5_ in its current form is however not cell permeable and therefore does not possess drug-like properties. The crystal structure of the phosphatase domain of SHIP-2 bound to BiPh(2,3′,4,5′,6)P_5_ has identified a flexible loop which folds over and encloses the ligand (Mills et al., 2012) and may have implications for development of small molecules that target SHIP-1. The targeting of this region may allow more SHIP-2 specific drugs to be developed. Cell permeable pan-SHIP-1/2 inhibitors have also recently been identified and have been reported to kill multiple myeloma cells (Fuhler et al., 2011). The development of SHIP-2 specific compounds suggests that SHIP-2 may be a potential target with which to treat a range of diseases, in addition to allowing the poorly understood role of SHIP-2 in the immune system, to be probed in greater depth.

## Summary

The difficulties of developing PI3Kγ inhibitors with sufficient selectivity over other PI3K isoforms has in part, led to the search for alternative drug targets to selectively modify PI3K signaling in the immune system. This search revealed the potential for exploiting the lipid phosphatase SHIP-1, an endogenous and leukocyte-restricted regulator of PI3K signaling. Small molecule regulators of this protein have shown early promise in inflammatory, transplantation, and cancer settings, and are currently in phase IIa clinical trials to evaluate the safety, tolerability, and pharmacokinetics (Table 1). The selectivity profile of compounds targeting SHIP-1 is at present quite limited and while they appear to exhibit specificity for SHIP-1 versus SHIP-2 and PTEN, it remains to be seen whether there are other off-target effects. Despite this early promise, the targeting of SHIP-1 (particularly with inhibitors), is not without its potential problems. For example, SHIP-1 deficiency leads to a number of pathologies including fibrotic lung disease (Rauh et al., 2005), osteoporosis (Moon et al., 2011), and the development of spontaneous intestinal inflammation and fibrosis (Kerr et al., 2011; McLarren et al., 2011). An important factor to consider is that targeting catalytic activity may not be sufficient to inhibit all SHIP-1 mediated effects, given that SHIP-1 also fulfills key non-catalytic scaffolding functions (Song et al., 2005; Peng et al., 2010; Bao et al., 2012; Ruschmann et al., 2012). Hence, small molecule-based strategies to target catalytic activity are unlikely to affect these non-catalytic functions. This may be beneficial on the one hand, if pathological consequences are dependent on catalytic functions as such approaches will likely retain the non-enzymatic functions and hopefully limit unwanted side-effects. On the other hand, such strategies may be ineffective if pathological consequences are driven by non-enzymatic functions of SHIP-1. It is interesting to note however, that prolonged inhibition of SHIP-1 with 3AC leads to proteasome-dependent degradation of SHIP-1 (Fuhler et al., 2011).

## Conflict of Interest Statement

The authors declare that the research was conducted in the absence of any commercial or financial relationships that could be construed as a potential conflict of interest.
